# Supplementary material for: Convergent Evolution Associated with the Loss of Developmental Diapause May Promote Extended Lifespan in Bees
Source: Genome Biol Evol. 2024 Nov 23;16(12):evae255. doi: 10.1093/gbe/evae255 (PMC11632380; doi:10.1093/gbe/evae255)
Supplement: evae255_Supplementary_Data [file evae255_supplementary_data.zip › Supplementary reorder.docx]

Supplementary table S1 → Supplementary table S1

Supplementary table S2 - Supplementary table S5

Supplementary table S3 - Supplementary table S6

Supplementary table S4 - Supplementary table S7

Supplementary table S5 - Supplementary table S8

Supplementary table S6 - Supplementary table S9

Supplementary table S7 - Supplementary table S2

Supplementary table S8 - Supplementary table S3

Supplementary table S9 - Supplementary table S4
